# Segmental correction of adolescent idiopathic scoliosis by all-screw fixation method in adolescents and young adults. minimum 5 years follow-up with SF-36 questionnaire

**DOI:** 10.1186/1748-7161-7-5

**Published:** 2012-02-19

**Authors:** Ching-Hsiao Yu, Po-Quang Chen, Shu-Chuang Ma, Chee-Huan Pan

**Affiliations:** 1 Department of Orthopaedic Surgery, Tao-Yuan General Hospital, No.1492, Zhongshan Rd, Taoyuan City 330, Taiwan, (R.O.C; 2 Department of Orthopaedic Surgery, National Taiwan University Hospital, No. 7. Chung San South Road, Taipei 100, Taiwan

**Keywords:** Adolescent idiopathic scoliosis (AIS), All-screw method, SF-36 questionnaire

## Abstract

**Background:**

In our institution, the fixation technique in treating idiopathic scoliosis was shifted from hybrid fixation to the all-screw method beginning in 2000. We conducted this study to assess the intermediate -term outcome of all-screw method in treating adolescent idiopathic scoliosis (AIS).

**Methods:**

Forty-nine consecutive patients were retrospectively included with minimum of 5-year follow-up (mean, 6.1; range, 5.1-7.3 years). The average age of surgery was 18.5 ± 5.0 years. We assessed radiographic measurements at preoperative (Preop), postoperative (PO) and final follow-up (FFU) period. Curve correction rate, correction loss rate, complications, accuracy of pedicle screws and SF-36 scores were analyzed.

**Results:**

The average major curve was corrected from 58.0 ± 13.0° Preop to 16.0 ± 9.0° PO(*p *< 0.0001), and increased to 18.4 ± 8.6°(*p *= 0.12) FFU. This revealed a 72.7% correction rate and a correction loss of 2.4° (3.92%). The thoracic kyphosis decreased little at FFU (22 ± 12° to 20 ± 6°, (*p *= 0.25)). Apical vertebral rotation decreased from 2.1 ± 0.8 PreOP to 0.8 ± 0.8 at FFU (Nash-Moe grading, *p *< 0.01). Among total 831 pedicle screws, 56 (6.7%) were found to be malpositioned. Compared with 2069 age-matched Taiwanese, SF-36 scores showed inferior result in 2 variables: physical function and role physical.

**Conclusion:**

Follow-up more than 5 years, the authors suggest that all-screw method is an efficient and safe method.

## Background

In the past decade, pedicle screws have become the mainstream of the fixation method used to correct spinal deformities. Surgical treatment of adolescent idiopathic scoliosis (AIS) with an all-screw method has also proven to be a effective and safe procedure [1-3].

Compared with other spinal fixation devises, pedicle screws have greater correction power because of their three-column fixation [4,5]. In our experience, the use of the all-screw method achieves a superior three-dimensional curve correction rate than the two previous methods: the all-hook method and hook/screw hybrid method [6,7] Additionally, more motion segments could be saved by reduction of the fusion length in thoracic scoliosis [1]. However, the insertion of pedicle screws in the thoracic spine is always a safety concern, especially in the upper thoracic area because of small pedicle calibers, close anatomical relations to adjacent neural structures, and deformed anatomical structures [8,9]. While it has been claimed that the insertion of pedicle screws, even with free-hand technique, is rather safe [2,8], complications such as nerve root injury and durotomy associated with screw placement have been reported [9,10]. As a result, the treatment of idiopathic scoliosis with posterior fusion and segmental instrumentation using either thoracic screws or lamina hooks remains contentious [7,11].

In our institution, the fixation technique in treating idiopathic scoliosis was shifted from hybrid fixation to the all-screw method beginning in 2000. We have observed an improved correction rate and minimal complications. We presume that all-screw construct could provide a good three-dimensional curve correction and maintenance.

The purposes of the current study were to evaluate the intermediate-term (> 5 years) effectiveness and safety profile of the all-screw fixation method in the treatment of idiopathic scoliosis. The functional outcome and quality of life was assessed by means of an established questionnaire.

## Methods

### Patients

The study was approved by the local ethics committee. Forty-nine AIS patients (38 females, 11 males) were retrospectively evaluated during a consecutive series from September 2001 to December 2003. Patients with congenital scoliosis, neuromuscular scoliosis and those who had undergone revision surgeries were excluded. The minimum follow-up was 5 years (mean, 6.1 years; range, 5.1-7.3 years). All patients were operated on by the senior surgeon (PQC) and the first author (CHY) in a single institution with the all-screw method. The mean patient age at the time of the operation was 18.5 ± 5.0 years (range, 11.7-31.4 years). The average Risser grade was 3.8 ± 1.4 grade (range, 2-5 grade). Most patients were treated for thoracic scoliosis. According to the King classification [12], the patient number were King type I (n = 10), King type II (n = 16), King type III (n = 20), King type IV (n = 1) and King type V (n = 2). Using the Lenke classification [13], the patient numbers were type 1 (n = 30), type 2 (n = 2), type 3 (n = 8), type 5 (n = 5), and type 6 (n = 4). Patient data were obtained from the medical records and radiographs (Table 1).

**Table 1:** Patient Demographics and Operative Data

Data	All	ADIS	AIS	*P *value
**Patient Numbers**	49	15	34	

Age at Operation* (years)	18.5(11.7-31.4)	25.0(20.0-31.4)	15.6(11.7-19.8)	< 0.001

Risser grade*	3.8 ± 1.4	5.0	3.2 ± 1.4	< 0.001

**King type (1:2:3:4:5)**	(10:16:20:1:2)	(1:2:10:0:2)	(9:14:10:1:0)	

**Lenke type (1:2:3:4:5:6)**	(30:2:8:0:5:4)	(10:2:2:0:1:0)	(20:0:6:0:4:6)	

**Op method (PSF: ASF + PSF)**	(42:7)	(14:1)	(28:6)	

Numbers of fused segments*	10.8(8-13)	10.9(8-14)	10.7(8-13)	0.566

Followup* (years)	6.05	6.62	5.8	0.476

*Data are expressed as mean, with range in parentheses; there were no significant differences between the two groups except in age of operation and Risser grade; *ADIS *adult-type adolescent idiopathic scoliosis

*AIS *adolescent idiopathic scoliosis; *PSF *posterior spinal fusion; *ASF *anterior spinal fusion

Patients were further divided in two groups, depending on the age of surgery: the adolescent (AIS) group (≤ 20 years, n = 34) and the young adult (ADIS) group (> 20 years, n = 15). The patient demographics in the AIS and ADIS groups were similar except the age of surgery and Risser grade (Table 1).

The mean age at surgery for the ADIS patients was 25.0 ± 3.2 years (range, 20.0 to 31.4 years) while those in the AIS patients was 15.6 ± 1.9 years (range, 11.7 to 19.8 years)(*p *< 0.05). The mean Risser grade for the ADIS group was 5.0 whereas for AIS group was 3.2 ± 1.4 (*p *< 0.05). The curve classifications was similar between two groups with thoracic curves were predominant.

### Procedures

Pedicle screws are inserted on the concave side one by one from the lumbar up to the thoracic segments. Then the screws are inserted to the convex side, ranging in every other segment from lower to the uppermost segment. We prefer inserting 2 screws each in the lower and uppermost adjacent 2 segments. At this stage, plain X-ray films are taken both in the AP and lateral views to confirm the exact positions of the screws in most of the patients. But in some patients, these films are taken after completion of the corrective procedures. The contoured rods are linked to the rods by vertical tapping on the rods into the heads of the screws on each side. Facetectomies are also performed in each segment. Derotation is started on the concave side first after completion of connecting rod to the screws. This procedure is finished after rotating the rod to the vertical position as seen from the top. Only mild distraction of the screws is necessary. Actually, due to segmental insertion of the screws, distraction procedure is not possible.

In the lower lumbar scoliotic ends, due to its convexity, compression between screws can be performed. Then the same procedure is performed on the convex side. One or 2 cross links are placed, one in the upper and the other in the lower part of the construct. With proper decortication of the laminae, the bone chips are placed on them. In our cases, we use cancellous allogenous bone graft taken from bone bank for fusion. Thoracoplasty is considered and carried out only when the rib protrusion is obvious and the angle of trunk rotation (ATR) is greater than 15°.

Among the 49 cases, 42 underwent a one-stage posterior spinal fusion (PSF) and seven underwent a two-stage operation; the latter consisted of an anterior spinal fusion followed by posterior spinal fusion (APSF) with instrumentation. Four patients had additional thoracoplasty. The average number of fused spinal segments was 10.8 (range, 8-13).

In this series, the screw system used included the Universal Posterior System (UPS; Aaxter, Taipei, Taiwan) for 45 cases, the Moss-Miami (DePuy Spine, Raynham, MA, USA) for 2 cases, and the Diapason (Stryker, Kalamazoo, MI, USA) system for the remaining 2 cases. The titanium-made UPS system has 6.5, 5.5 and 4.5 mm diameter screws of different lengths. The rod was 6.0 mm diameter with a smooth surface. As much as possible, mono-axial screws were used because the correction power can reach from posterior bony part to the anterior body portion [14].

Apical trunk rotation (ATR) was presently assessed using the forward bending test at preoperative (PreOP) and final follow-up (FFU) period. The three parts of the ATR were recorded including thoracic level (TH), thoracolumbar level (TL) and lumbar level (L).

Spine radiographic assessment included standing postero-anterior and lateral radiographs at PreOP), 1 week postoperative (PO) and at FFU. The passive standing lateral bending films were taken before surgery to measure curve flexibility. Three-dimensional deformity of each patient was assessed. Major and minor curves on the coronal plane were measured by the Cobb method. The sagittal alignment included the thoracic kyphosis from T4 to T12, and lumbar lordosis from L1 to L5. Rotation of the apical vertebra was determined by the Nash and Moe method on standing postero-anterior films [15]. The rotational correction of the apex of the curve was assessed by comparing the PreOP and PO rotational deformity of the apical vertebra. In cases where the pedicle of the apical vertebra was obscured by the inserted pedicle screw, the rotations of adjacent uninstrumented vertebral (superiorly or inferiorly) were compared. There are no cases in which the apical vertebrae and adjacent ones instrumented with pedicle screws which obscure the pedicle. The curve correction rate of all three planes, loss of correction rate and numbers of fused segments were analyzed to evaluate the effectiveness and maintenance of operation. Complications and malposition rates of the pedicle screws were documented to ascertain the safety profile. The positions of pedicle screws at every instrumented vertebral level in the 1-week PO postero-anterior and lateral radiographs were studied. This was performed by an independent spinal surgeon who was not involved with this study. The outcome of the evaluations was assigned as intrapedicular or extrapedicular placement. Extrapedicular placement was further categorized into inferior, superior, lateral or medial position. Functional outcome was assessed using the SF-36 questionnaire (Taiwan version 1.0) [16] at the latest follow-up. Because of the lack of preoperative data, we also compared this data to an age-matched population of 2069 individuals in Taiwan [16]. We also separate the results by AIS and ADIS patients. The age at follow-up is averaged 24.2 ± 4.5 years (range, 17.2-36.6 years) while 2 patients were aged under 18 years at follow-up

### Statistical analysis

The *T*-test was used with level of significance of 5% (*p *< 0.05) to compare the differences between PreOP, PO and FFU data.

## Results

### Coronal plane correction

In the coronal plane, the PreOP Cobb angle of the major curve was 58.0 ± 13.0° (range, 40-107), which was corrected by 72.7% to 16.0 ± 9.0° (range, 5-51) (*P *< 0.0001)after surgery (Table 1). The PreOP Cobb angle of the compensatory curve was 38 ± 10° (range, 20-64), which was corrected by 72.4% to 11 ± 8° (range, 0-26) (*P *< 0.001) after surgery. The mean correction loss for major curve of all 49 patients at FFU was 2.4° (3.92%) (Table 2).

**Table 2:** Coronal and Sagittal Parameters Results

	Preop (°)	PO (°)		FFU (°)		Flexibility	CR	CLS
**Major curve**	58.0 ± 13.0	16.0	± 9.0*	18.4	± 8.6	41.8%	72.7%	3.92%
	
		(*p *< 0.0001)		(*p *= 0.12)				

**Thoracic Kyphosis**	22.0 ± 12.0	18.0	± 7.0*	20.0	± 6.0			
	
		(*p *< 0.001)		(*p *= 0.25)				

**Lumbar Lordosis**	37.0 ± 12.0	33.0	± 10.0*	37.0	± 12.0**			
	
		(*p *< 0.001)		(*p *< 0.005)				

data are expressed as mean ± standard deviation;*significantly different from preoperative data; ** significantly different from postoperative data

*Preop *preoperative, *PO *postoperative, *FFU *final follow-up, *CR *correction rate, *CLS *correction loss rate

$$\text{Correction rate = }\begin{array}{c} \text{Preop angle - Postop angle}\\ ---------------\\ \text{Preop angle} \end{array}\times 100\%;\text{ Correction loss = }\begin{array}{c} \text{Final follow - up angle - Postop angle}\\ --------------------\\ \text{Prop angle} \end{array}\times 100\%$$

### Sagittal curve correction

The mean preoperative thoracic kyphosis (T4-T12) of 22 ± 12° (range, 0-50) was reduced to 18 ± 7° (range, 3-37) (*P *< 0.001) after surgery and to 20 ± 6° (range, 5-35) (*P *= 0.12) at FFU. The mean preoperative lumbar lordosis (L1-L5) was decreased from 37 ± 12° (range, 60-5) to 33 ± 10°(range, 63-12) (*P *< 0.001) after surgery, and to 37 ± 12° (range, 63-12) (*P *< 0.005) at FFU (Table 2).

Ten out of the 49 patients had thoracic hypokyphosis preoperatively (≤ 10°). They achieved better kyphotic alignment after the index procedure (mean, from 6.5 ± 3.5° to 12.8° ± 5.3). (*P *< 0.005). The mean angle improved to 18.6 ± 6.9° degrees at FFU (*P *< 0.005). Of these 49 patients, four had thoracic hyperkyphosis (≥ 40). The mean preoperative thoracic kyphosis angle of 45.8 ± 3.0° (range, 43-50) was reduced to 24.0 ± 9.0° (range, 15-36) (*P *< 0.001)after the operation and to 22.0 ± 1.8° (range, 20-24) (*P *< 0.005) at FFU.

### Axial plane correction

The mean preoperative ATR was reduced from 7.8 ± 5.0° to 5.6 ± 3.8° (TH level, *P *= 0.28), from 8.8 ± 5.0° to 6.3 ± 4.1° (TL level, *P *= 0.15), and from 8.9 ± 5.2° to 3.1 ± 3.5° (L level, *P *< 0.001) at FFU. The mean Nash and Moe grade of PreOP apical vertebra rotation of 2.1 ± 0.8 was improved to 0.7 ± 0.8(*P *< 0.01), but increased to 0.8 ± 0.8 at FFU(*p *= 0.45) (Table 3).

**Table 3:** Axial l Plane Parameters Results

APR (Nash Moe method)			ATR (forward bending test)		
**Preop**	**PO**	**FFU**		**Preop (°)**	**FFU (°)**

2.1 ± 0.8	0.7 ± 0.8*	0.8 ± 0.8	TH	7.8 ± 5.0	5.6 ± 3.8

	(*p *< 0.001)	(*p *= 0.45)			(*p *= 0.28)

			TL	8.8 ± 5.0	6.3 ± 4.1

					(*p *= 0.15)

			L	8.9 ± 5.2	3.1 ± 3.5*

					(*p *< 0.001)

data are expressed as mean ± standard deviation;*significantly different from preoperative data

*APR *apical vertebral rotation; *ATR *apical trunk rotation

*Preop *preoperative, *PO *postoperative, *FFU *final follow-up, *TH *thoracic, *TL *thoracolumbar, *L *lumbar

### Insertion accuracy of pedicle screws

A total of 831 pedicle screws were inserted, which included 596 thoracic and 235 lumbar screws. The mean number of pedicle screws inserted per patient was 17 (range, 10-24). Among the 831 pedicle screws, 56 (6.7%) were found to be malpositioned and most of them were situated in the thoracic spine (42/56, 75%). Furthermore, 16 screws were found inferiorly, 25 found laterally, and 15 superiorly to the pedicle. None of them were found medially. Fortunately, there were no patients who sustained a nerve root injury during surgery.

### Complications

There were no neurological deficits, visceral or vascular injuries after the operation and during the admission course. Also, there were no instrumentation failures or pedicle screws pull-out at the latest follow-up. One patient developed an anesthesia complication of right hemopneumothorax after central venous line insertion. The hemopneumothorax resolved uneventfully after chest tube insertion.

### Short-form 36 (SF-36) questionnaire findings

**Figure 1:**
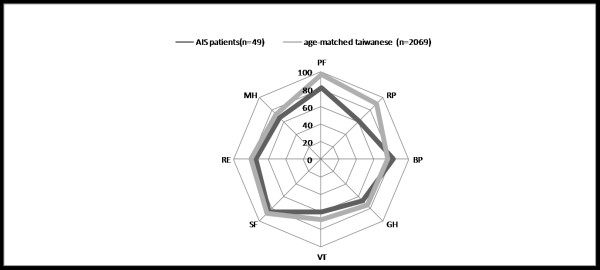
**Comparison of SF-36 scores between 49 AIS patients (black line) and 2069 age-matched Taiwanese (grey line) showed lower scores in PF and RP variables of AIS patients**. PF = Physical Function; RP = Role Physical; BP = Bodily Pain; GH = General Health; VT = Vitality scale; SF = Social Function; RE = Role Emotional; MH = Mental Health.

All 49 patients completed the SF-36 questionnaire. We calculated their scores by using the scoring algorithms and data entry criteria provided by the SF-36 Health Survey Manual. The scores were then averaged to develop norms in each category: Physical Functioning scale (PF) = 81.94, Role Physical scale (RP) = 61.29, Bodily Pain scale (BP) = 82.42, General Health (GH) = 67.26, Vitality scale (VT) = 60.48, Social Functioning scale (SF) = 85.08, Role Emotional scale (RE) = 75.01, and Mental Health scale (MH) = 67.59. The Physical Component Summary (PCS) was 48.49 ± 6.52 while the Mental Component Summary (MCS) was 48.32 ± 7.03 (Table 4). Compared with the 2069 age-matched Taiwanese, we found significantly lower SF-36 scores of these AIS patients in two variables: physical function (81.94 versus 96.82), and role physical (61.29 versus 89.63) (Figure 1). We further compared the SF-36 scores between AIS and ADIS groups (Table 5). Among them, AIS group had non-significant lower scores in two variables: role physical scale (53.75 versus 75.00, *P *= 0.76) and role emotional scale (69.99 versus 84.13, *P *= 0.63). The Physical Component Summary (PCS) and Mental Component Summary (MCS) between these two groups were similar. (PCS: 48.29 versus 49.96, *P *= 0.26, MCS: 48.80 versus 47.45, *P *= 0.65)

**Table 4:** SF-36 Scores of 49 AIS Patients

SF-36 Variables	Mean	St. Deviation
**PF**	81.94 (35 ~ 100)	12.29

**RP**	61.29 (0 ~ 100)	32.17

**BP**	82.42(22 ~ 100)	18.96

**GH**	67.26(25 ~ 100)	18.36

**VT**	60.48(10 ~ 100)	17.62

**SF**	85.08(37.5 ~ 100.0)	14.22

**RE**	75.01(0 ~ 100)	35.31

**MH**	67.59 (28 ~ 92)	15.01

**PCS**	48.89(29.3 ~ 61.0)	6.52

**MCS**	48.32(26 ~ 58)	7.03

Data are expressed as mean, with range in parentheses

*PF *Physical Functional scale; *RP *Role Physical scale; *BP *Bodily Pain scale

*GH *General Health scale; *VT *Vitality scale; *SF *Social Functioning scale

*RE *Role Emotional scale; *MH *Mental Health scale

*PCS *Physical Component Summary, *MCS *Mental Component Summary

**Table 5:** Comparision of SF-36 Scores Between AIS and ADIS Pateints

	PF	RP	BP	GH	VT	SF	RE	MH	PCS	MCS
**AIS (n = 34)**	82.50	53.75	83.05	68.45	61.00	86.25	69.99	71.15	48.29	48.80

**ADIS(n = 15)**	80.91	75.00	81.27	65.09	59.55	82.96	84.13	61.13	49.96	47.45

***P *value**	0.83	0.76	0.78	0.75	0.69	0.95	0.63	0.34	0.26	0.65

*AIS *adolescent idiopathic scoliosis; *ADIS *adult-type adolescent idiopathic scoliosis

Data are expressed as mean *PF *Physical Functional scale; *RP *Role Physical scale; *BP *Bodily Pain scale

*GH *General Health scale; *VT *Vitality scale; *SF *Social Functioning scale

*RE *Role Emotional scale; *MH *Mental Health scale

*PCS *Physical Component Summary, *MCS *Mental Component Summary

## Discussion

Although some controversy does exist, the all- screw construct has gained worldwide popularity in treating idiopathic scoliosis in the last decade. This reflects the majority consensus that the all pedicle screw approach can achieve better three-dimensional correction and less correction loss compared to the all-hook and hybrid construct approaches.

**Figure 2:**
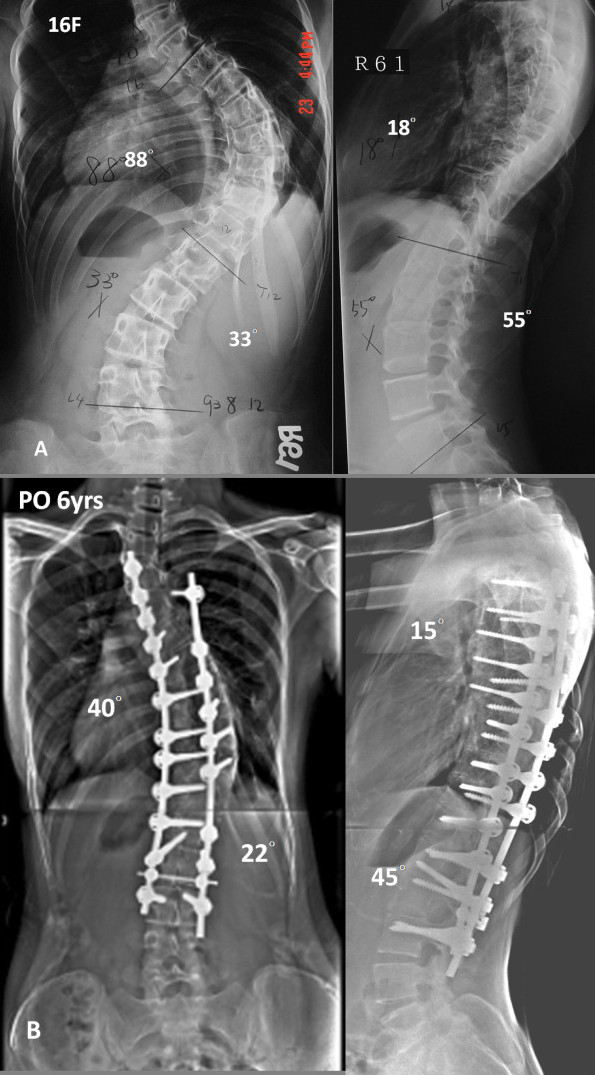
**A Preoperative AP, and lateral radiographs of a 13 year-old-female with 1A curve**. B, The patient underwent anterior fusion followed by posterior spinal fusion from T3 to L3 with all-screw construct. Postoperative radiographs of 6 years followup showed 55% correction rate.

A direct comparison between the hybrid and all-screw construct revealed superior major curve correction in all-screw group (70% versus 56%) in 58 AIS patients [7]. This is consistent with a previous study using the Moss-Miami hybrid construct in 61 AIS patients, which revealed a 56% correction rate of the major thoracic curve [17]. In the present study, a correction rate of 72.7% was achieved on the coronal plane with a flexibility of 41.8% (Figure 2). This correction rate echoes those of other studies on segmental pedicle screw fixation of idiopathic scoliosis [1,3,7].

Correction loss of the major curve with various types of instrumentation has been given great attention in the literature. Improvements in the fixation instruments used in scoliosis surgery have improved the correction loss rate. In one study, for example, the use of CD instruments in 64 patients produced an average correction loss for King type 2 and 3 curves of 5.2° (11.6%) and 3.9° (8.3%), respectively [18]. The use of pedicle screws, which can provide better holding power, can substantially improve the correction loss rate from 3-6% [3,7]. In this series with minimum 5 year follow-up, the correction loss averaged 2.4° (3.9%). Compared to our own previous reports with 7.8% correction loss rate for all-hook and 6.6% for hybrid construct [19], we presently obtained a better result. Also, this compares favorably with other intermediate-term studies using segmental screw instrumentation [1].

The results of long-term follow-up of Harrington rod instrumentation for correction of idiopathic scoliosis highlights the importance of preserving or restoring sagittal alignment of the spine PO. Numerous reports have pointed out the complications of significant loss of lumbar lordosis, which results in "flat-back syndrome" [20]. In this study, the thoracic kyphosis and lumbar lordosis were well-maintained PO and at the latest follow-up. In addition, the thoracic hypokyphosis and hyperkyphosis in our 14 patients improved to a better kyphotic angle. The same effect has been reported in a screw group with good correction of preoperative hypokyphosis (from 9° to 27°) [6], which was comparable to the hook or hybrid groups. In another study, the PreOP hyperkyphotic status in 26 patients was reduced in the screw group [21].

Safety on insertion of thoracic pedicle screws is always a great concern [2], especially at the concave upper thoracic segment with the deformed and small caliber pedicles. Although the incidence is rare, the complications of thoracic screw misplacement can be obvious and fatal. Reported complication rates associated with pedicle screw placement range from 0-25% [10], in which the malposition of the screw is the most common problem. In addition, major complications such as neurologic or vascular injury are exceptionally rare. In our series, the screw misplacement rate was 6.7%. Also, there were no major or fatal complications. Therefore, we consider that placement of thoracic pedicle screws using free hand technique is a safe and reliable technique. But, the technique requires experienced personal with better tactile feedback skills and a thorough understanding of the deformed spine anatomy. The technique additionally benefits from the more user-friendly instrumentation tools developed in recent years. These included a smaller pedicle finder (2 mm) and various choices of pedicle screws for upper thoracic segments (25 mm, 30 mm, and 35 mm long). While we did not use the navigation system for pedicle screw placement, we think this modern modality could be helpful for deformity operations.

Short form-36 (SF-36) and Scoliosis Research Society (SRS) Outcomes Instrument (SRS-22) are both well-validated questionnaires of "quality of life" assessment [22,23]. Although a Chinese adaptation of the SRS-22 has been previously validated in Hong Kong, no validation exists for use in Taiwan. SF-36, on the other hand, has been validated and used in Taiwan since 1996 (Taiwan version 1.0) [24]. The SF-36 health status questionnaire is a patient self-perceived tool and has been documented in several studies when assessing functional status of scoliosis patients [22,23]. Physical and mental status is also very crucial for these young patients, who are usually students or young employees with many physical and social activities. In the present study, there were two significantly lower scores compared to age-matched Taiwanese including physical function (PF), and role physical (RP). It is notable that both variables belonged to the physical health category. This indicates that, even with similar mental health compared with healthy populations, such patients subjectively demonstrate inferior physical status and role limitation. This is consistent with a Norway study with 30 AIS patients [25].

Limitations of this study include relatively small sample sizes and no comparative groups. We also did not use CT scan to assess the accuracy of screw placement. However, this study demonstrates the early experience and clinical results of using all-screw method by a single surgeon in a single institution.

## Conclusions

Based upon the results obtained from this review of 49 AIS patients with minimum 5 years of follow-up, we suggest that the all-screw method is efficient and safe. The outcomes in three-dimensional correction are satisfactory and comparable. The curve maintenance is good with minimal loss of correction. Compared with age-matched healthy populations, these patients did present some limitations on their physical health, as assessed by the SF- 36 questionnaire.

## Competing interests

One or more of the authors have received funding from NSC Grant

NSC-2622 B 002-002 (PQC) and the Taiwan Spine Research Foundation.

## Authors' contributions

CHY, SCM and CHP participated in the design of the study and performed the statistical analysis. CHY and PQC conceived of the study, and participated in its design and coordination and helped to draft the manuscript. All authors read and approved the final manuscript

## References

[B1] Suk SI Lee SM Chung ER Kim JH Kim SS Selective thoracic fusion with segmental pedicle screw fixation in the treatment of thoracic idiopathic scoliosis: more than 5-year follow-up Spine 2005 30 14 1602 1609 10.1097/01.brs.0000169452.50705.61 16025028

[B2] Suk SI Kim WJ Lee SM Kim JH Chung ER Thoracic pedicle screw fixation in spinal deformities: are they really safe? Spine 2001 26 18 2049 2057 10.1097/00007632-200109150-00022 11547207

[B3] Lehman RA Jr Lenke LG Keeler KA Kim YJ Buchowski JM Cheh G Kuhns CA Bridwell KH Operative treatment of adolescent idiopathic scoliosis with posterior pedicle screw-only constructs: minimum three-year follow-up of one hundred fourteen cases Spine 2008 33 14 1598 1604 10.1097/BRS.0b013e318178872a 18552676

[B4] Lehman RA Jr Polly DW Jr Kuklo TR Cunningham B Kirk KL Belmont PJ Jr Straight-forward versus anatomic trajectory technique of thoracic pedicle screw fixation: a biomechanical analysis Spine 2003 28 18 2058 2065 10.1097/01.BRS.0000087743.57439.4F 14501914

[B5] Lehman RA Jr Kuklo TR Use of the anatomic trajectory for thoracic pedicle screw salvage after failure/violation using the straight-forward technique: a biomechanical analysis Spine 2003 28 18 2072 2077 10.1097/01.BRS.0000084628.37133.BA 14501916

[B6] Suk SI Lee CK Kim WJ Chung YJ Park YB Segmental pedicle screw fixation in the treatment of thoracic idiopathic scoliosis Spine 1995 20 12 1399 1405 7676339

[B7] Kim YJ Lenke LG Kim J Bridwell KH Cho SK Cheh G Sides B Comparative analysis of pedicle screw versus hybrid instrumentation in posterior spinal fusion of adolescent idiopathic scoliosis Spine 2006 31 3 291 298 10.1097/01.brs.0000197865.20803.d4 16449901

[B8] Smorgick Y Millgram MA Anekstein Y Floman Y Mirovsky Y Accuracy and safety of thoracic pedicle screw placement in spinal deformities J Spinal Disord Tech 2005 18 6 522 526 10.1097/01.bsd.0000154448.90707.a8 16306843

[B9] Papin P Arlet V Marchesi D Rosenblatt B Aebi M Unusual presentation of spinal cord compression related to misplaced pedicle screws in thoracic scoliosis Eur Spine J 1999 8 2 156 159 10.1007/s005860050147 10333156 PMC3611145

[B10] Di Silvestre M Parisini P Lolli F Bakaloudis G Complications of thoracic pedicle screws in scoliosis treatment Spine 2007 32 15 1655 1661 10.1097/BRS.0b013e318074d604 17621214

[B11] Jansen RC van Rhijn LW Duinkerke E van Ooij A Predictability of the spontaneous lumbar curve correction after selective thoracic fusion in idiopathic scoliosis Eur Spine J 2007 16 9 1335 1342 10.1007/s00586-007-0320-3 17294054 PMC2200742

[B12] King HA Moe JH Bradford DS Winter RB The selection of fusion levels in thoracic idiopathic scoliosis J Bone Joint Surg Br 1983 65 9 1302 1313 6654943

[B13] Lenke LG Betz RR Harms J Bridwell KH Clements DH Lowe TG Blanke K Adolescent idiopathic scoliosis: a new classification to determine extent of spinal arthrodesis J Bone Joint Surg Br 2001 83-A 8 1169 1181 11507125

[B14] Lonner BS Auerbach JD Boachie-Adjei O Shah SA Hosogane N Newton PO Treatment of thoracic scoliosis: are monoaxial thoracic pedicle screws the best form of fixation for correction? Spine 2009 34 8 845 851 10.1097/BRS.0b013e31819e2753 19365255

[B15] Nash CL Jr Moe JH A study of vertebral rotation J Bone Joint Surg Br 1969 51 2 223 229 5767315

[B16] Lu J-FR Tseng H-M Tsai Y-J Assessment of Health-related Quality of Life in Taiwan (I): Development and Psychometric Testing of SF-36 Taiwan Version Taiwan Journal of Public Health 2003 22 6 501 511

[B17] Chen PQ Management of scoliosis J Formos Med Assoc 2003 102 11 751 761 14724720

[B18] Puno RM Grossfeld SL Johnson JR Holt RT Cotrel-Dubousset instrumentation in idiopathic scoliosis Spine 1992 17 8 Suppl S258 S262 1523509 10.1097/00007632-199208001-00008

[B19] Chen PQ Yang SH Surgical Correction of Adolescent Idiopathic Scliosis: A 5 to 12 years follow-up study of thoracic type adolescent idiopahtic scoliosis undergoing Cotrel-Dubusset instrumentation Journal of Bone and Joint Surgery - British Volume 2002 84-B SUPP_III 239

[B20] Lagrone MO Bradford DS Moe JH Lonstein JE Winter RB Ogilvie JW Treatment of symptomatic flatback after spinal fusion J Bone Joint Surg Br 1988 70 4 569 580 3356724

[B21] Kim YJ Lenke LG Cho SK Bridwell KH Sides B Blanke K Comparative analysis of pedicle screw versus hook instrumentation in posterior spinal fusion of adolescent idiopathic scoliosis Spine 2004 29 18 2040 2048 10.1097/01.brs.0000138268.12324.1a 15371706

[B22] Lai SM Asher M Burton D Estimating SRS-22 quality of life measures with SF-36: application in idiopathic scoliosis Spine 2006 31 4 473 478 10.1097/01.brs.0000200049.94329.f4 16481961

[B23] Schwab F Dubey A Pagala M Gamez L Farcy JP Adult scoliosis: a health assessment analysis by SF-36 Spine 2003 28 6 602 606 12642769 10.1097/01.BRS.0000049924.94414.BB

[B24] Lee Y-H Yang N-P Wei K-Y Chou P Comparison of quality of life between subjects with traumatic wrist and hip fractures Changhua J Med 2005 10 51 58

[B25] Kibsgard T Brox JI Reikeras O Physical and mental health in young adults operated on for idiopathic scoliosis J Orthop Sci 2004 9 4 360 363 15278773 10.1007/s00776-004-0798-z

